# PmrC (EptA) and CptA Negatively Affect Outer Membrane Vesicle Production in Citrobacter rodentium

**DOI:** 10.1128/JB.00454-18

**Published:** 2019-03-13

**Authors:** Anshul Sinha, Sammy Nyongesa, Charles Viau, Samantha Gruenheid, Frédéric J. Veyrier, Hervé Le Moual

**Affiliations:** a Department of Microbiology and Immunology, McGill University, Montreal, Quebec, Canada; b INRS-Institut Armand-Frappier, Bacterial Symbionts Evolution, Laval, Quebec, Canada; c Faculty of Dentistry, McGill University, Montreal, Quebec, Canada; Université de Montréal

**Keywords:** *Citrobacter rodentium*, LPS modifications, outer membrane vesicles, envelope stress response, oxidative stress, two-component regulatory systems

## Abstract

Outer membrane vesicles (OMVs) are naturally produced by Gram-negative bacteria by a bulging of the outer membrane (OM) and subsequent release into the environment. By serving as vehicles for various cargos, including proteins, nucleic acids and small metabolites, OMVs are central to interbacterial interactions and both symbiotic and pathogenic host bacterial interactions. However, despite their importance, the mechanism of OMV formation remains unclear. Recent evidence indicates that covalent modifications of lipopolysaccharides (LPS) influence OMV biogenesis. Several enteric bacteria modify LPS with phosphoethanolamine (pEtN) using the iron-regulated PmrC (EptA) and CptA pEtN transferases. In wild-type Citrobacter rodentium, the presence of increasing subtoxic concentrations of iron was found to stimulate OMV production 4- to 9-fold above baseline. C. rodentium uses the two-component system PmrAB to sense and adapt to environmental iron. Compared to the wild type, the C. rodentium Δ*pmrAB* strain exhibited heightened OMV production at similar iron concentrations. PmrAB regulates transcription of *pmrC* (also known as *eptA*) and *cptA*. OMV production in strains lacking either *pmrC* (*eptA*) or *cptA* was similarly increased in comparison to that of the wild type. Importantly, plasmid complementation of C. rodentium strains with either *pmrC* (*eptA*) or *cptA* resulted in a drastic inhibition of OMV production. Finally, we showed that β-lactamase and CroP, two enzymes found in the C. rodentium periplasm and outer membrane (OM), respectively, are associated with OMVs. These data suggest a novel mechanism by which C. rodentium and possibly other Gram-negative bacteria can negatively affect OMV production through the PmrAB-regulated genes *pmrC* (*eptA*) and *cptA*.

**IMPORTANCE** Although OMVs secreted by Gram-negative bacteria fulfill multiple functions, the molecular mechanism of OMV biogenesis remains ill defined. Our group has previously shown that PmrC (also known as EptA) and CptA maintain OM integrity and provide resistance to iron toxicity and antibiotics in the murine pathogen Citrobacter rodentium. In several enteric bacteria, these proteins modify the lipid A and core regions of lipopolysaccharide with phosphoethanolamine moieties. Here, we show that these proteins also repress OMV production in response to environmental iron in C. rodentium. These data support the emerging understanding that lipopolysaccharide modifications are important regulators of OMV biogenesis in Gram-negative bacteria.

## Abstract (precis)

Although OMVs secreted by Gram-negative bacteria fulfill multiple functions, the molecular mechanism of OMV biogenesis remains ill defined. Our group has previously shown that PmrC (also known as EptA) and CptA maintain OM integrity and provide resistance to iron toxicity and antibiotics in the murine pathogen Citrobacter rodentium. In several enteric bacteria, these proteins modify the lipid A and core regions of lipopolysaccharide with phosphoethanolamine moieties. Here, we show that these proteins also repress OMV production in response to environmental iron in C. rodentium. These data support the emerging understanding that lipopolysaccharide modifications are important regulators of OMV biogenesis in Gram-negative bacteria.

## INTRODUCTION

Outer membrane vesicles (OMVs) are spherical lipid structures of approximately 20 to 300 nm in size that are shed from the surface of all Gram-negative bacteria (1, 2). OMVs contain various cargos, including periplasmic and outer membrane (OM) proteins, toxins, enzymes, signaling molecules, lipopolysaccharides (LPS), DNA, and RNA (3–6). Due to their ability to carry these cargos over distances, OMVs were attributed multiple functions in both host-bacterial and interbacterial interactions. During infection, vesicles can be taken up by host cells and induce apoptosis through the release of toxins and enzymes (7). The pathogen-associated molecular patterns LPS and peptidoglycans carried by OMVs are recognized by pattern recognition receptors to initiate the host immune response (8, 9). OMVs can also provide defense against environmental insults by acting as decoys that prevent antibiotics, antimicrobial peptides (AMPs), and bacteriophages from reaching the bacterial cell (10). In addition, OMVs are involved in various interbacterial interactions. They facilitate predatory activity by delivering active enzymes to adjacent bacteria to aid in the establishment of ecological niches (11). Also, OMVs have been shown to carry quorum-sensing autoinducers and genetic material to facilitate interbacterial communication and horizontal gene transfer, respectively (4, 12–14).

Although many biological functions have been described for OMVs, the exact mechanism of OMV biogenesis remains unclear. It was first proposed that OMVs are produced when cross-links between specific lipoproteins of the outer membrane (OM) and the underlying peptidoglycan dissociate, resulting in OM bulges that pinch off from the cell envelope (15). Other studies have shown that OMV production may rely on the presence of certain molecules that induce membrane curvature in the OM. For example, the intercalation of the quorum-sensing molecule 2-heptyl-3-hydroxy-4-quinolone (PQS) into the Pseudomonas aeruginosa OM is thought to induce membrane curvature and subsequent vesicle budding (16). Another study proposed that the presence of phospholipids in the outer leaflet of the OM results in OMV formation (17). Other evidence suggests that OMV production is an adaptive response to environmental changes. Envelope and oxidative stresses, as well as the presence of AMPs or antibiotics, have been shown to result in increased vesiculation in several Gram-negative species (10, 18, 19). It was also proposed that OMVs are secreted to rid the cell envelope of damaged material (20, 21).

LPS is an important component of the OM that acts as a permeability barrier (22). LPS can be covalently modified in response to environmental cues to contribute additional protection to the OM against iron toxicity, antibiotics, and AMPs (23). These LPS modifications are regulated by the PmrAB and PhoPQ two-component systems (TCS). The PmrAB TCS is activated by the presence of ferric iron (Fe^3+^) and mildly acidic pH, while PhoPQ is activated by low concentrations of Mg^2+^, mildly acidic pH, and AMPs (24–26). In Salmonella enterica and Escherichia coli, the PhoPQ and PmrAB pathways are interconnected. PhoPQ controls PmrAB by regulating expression of the *pmrD* gene, which prevents dephosphorylation and inactivation of the PmrA response regulator (27). In S. enterica, PmrAB regulates the addition of 4-amino-4-deoxy-l-arabinose (l-Ara4N) to lipid A by the *arn* operon (28). It also regulates the addition of phosphoethanolamine (pEtN) to the lipid A and core portions of LPS by PmrC (also known as EptA) and CptA, respectively (29, 30). These modifications reduce the LPS negative charge and, in turn, provide increased resistance to AMPs and iron-induced oxidative stress (28–31). PhoPQ regulates expression of the PagL protein, which is responsible for deacylation of lipid A in S. enterica. PagL-mediated lipid A deacylation has been found to induce OMV production by promoting positive curvature of the OM (32).

Citrobacter rodentium is a natural murine pathogen that causes transmissible murine colonic hyperplasia (33). It is used as a surrogate model to study the human diarrheal pathogens enterohemorrhagic E. coli (EHEC) and enteropathogenic E. coli (EPEC). The defining feature of these pathogens is the formation of attaching and effacing (A/E) lesions during colonization of the gut mucosa. These A/E lesions are characterized by adherence to intestinal epithelial cells, formation of a pedestal, and effacement of brush-border microvilli (33, 34). Unlike S. enterica and E. coli, C. rodentium lacks the *pmrD* gene and, therefore, the PhoPQ TCS does not interfere with expression of PmrAB-regulated genes (31). Furthermore, C. rodentium lacks the PmrAB-regulated *arn* operon that is responsible for l-Ara4N addition to lipid A (31). We previously showed that the PmrAB-regulated genes *pmrC* (*eptA*) and *cptA* are important in maintaining OM integrity. Strains lacking *pmrAB*, *pmrC* (*eptA*), or *cptA* exhibited increased susceptibility to iron toxicity and antibiotics and increased OM permeability (31).

Environmental iron is an important contributor to bacterial oxidative stress. In the Fenton reaction, hydrogen peroxide is converted to a more reactive hydroxyl radical through the oxidation of Fe^2+^ into Fe^3+^ (35). These reactive hydroxyl radicals are highly damaging to lipid membranes, proteins, and nucleic acids (35). In this study, we explored the hypothesis that environmental iron and the iron-induced genes *pmrC* (*eptA*) and *cptA* affect OMV biogenesis in C. rodentium. We showed that production of OMVs is greatly increased in C. rodentium strains lacking the PmrAB TCS or the *pmrC* (*eptA*) and *cptA* genes. These results may further relate LPS modifications to OMV biogenesis and reveal a new role for PmrC (EptA) and CptA in C. rodentium.

## RESULTS

### The PmrAB TCS negatively affects OMV production.

**FIG 1:**
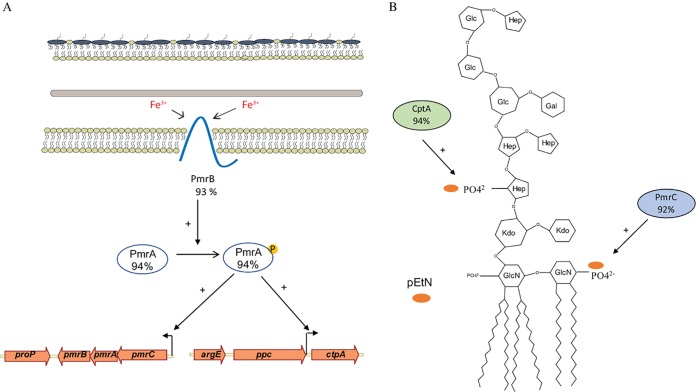
PmrAB regulates *pmrC* (*eptA*) and *cptA* in C. rodentium. (A) Model representing the PmrAB TCS. Under conditions of high Fe^3+^, the cytoplasmic PmrA response regulator becomes phosphorylated and active, resulting in differential expression of several genes, including *pmrC* (*eptA*) and *cptA* (31). (B) Schema showing PmrC (EptA) and CptA-mediated modifications of LPS. In E. coli, PmrC (EptA) and CptA add pEtN groups to the lipid A and core regions of LPS, respectively (36). Percentages refer to the similarity of the PmrA, PmrC (EptA), and CptA proteins between E. coli and C. rodentium.

**FIG 2:**
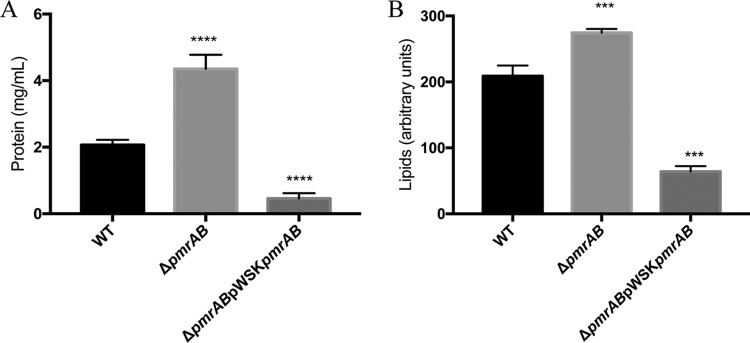
Deletion of the *pmrAB* TCS genes results in increased OMV biogenesis in C. rodentium. OMVs isolated from the C. rodentium wild-type, Δ*pmrAB*, and Δ*pmrAB* pWSK*pmrAB* strains grown in the presence of 50 μM FeCl_2_ were quantified by measuring protein and lipid contents (A) Protein content of OMVs was measured using the DC protein assay. Protein concentration was calculated using BSA as a standard. (B) The lipid content of OMV samples was determined by using the lipophilic dye FM 1-43. Cultures were grown to mid-log phase and normalized to an OD_595_ of 0.5 prior to OMV isolation. Values shown are the means ± standard error from three independent experiments. Significance was assessed relative to wild-type using one-way ANOVA; ****, *P* ≤ 0.0001; ***, *P* ≤ 0.001.

C. rodentium and related Enterobacteriaceae use the PmrAB TCS to sense and respond to environmental iron and modify the OM (Fig. 1A) (36). The role of PmrAB in OMV production was investigated by measuring the amounts of OMVs produced by the C. rodentium Δ*pmrAB* strain in the presence of FeCl_2_ (PmrAB-activating conditions). OMVs were isolated as described in the Materials and Methods. OMV production was measured using the detergent-compatible (DC) protein assay, which measures the total protein content of solubilized OMVs, and by the fluorescent probe FM1-43, which measures the lipid content of biological material with intact membranes. In a comparison of OMV production between the wild-type and Δ*pmrAB* strains grown at 50 μM FeCl_2,_ the Δ*pmrAB* strain exhibited a modest but significant increase in vesicle production, as measured by protein and lipid content (Fig. 2). Complementation- mediated overexpression of *pmrAB* resulted in drastic decreases of OMV production below wild-type levels (Fig. 2). Similar trends were observed with C. rodentium grown at various concentrations of FeCl_2_ (see Fig. S1 in the supplemental material). Collectively, these results indicate that the PmrAB TCS negatively affects OMV production in the presence of environmental iron.

### PmrC (EptA) and CptA negatively affect OMV production.

**FIG 3:**
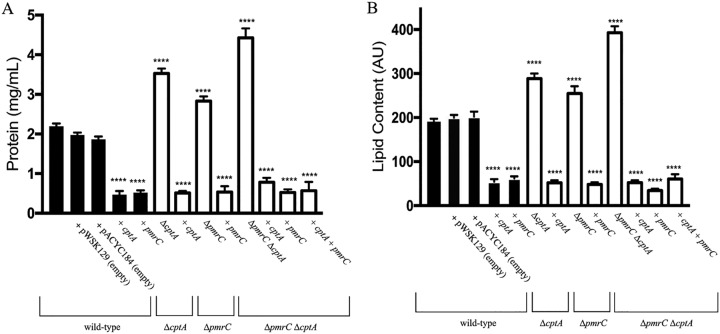
Deletion of *pmrC* (*eptA*) and *cptA* results in increased OMV biogenesis in C. rodentium. C. rodentium strains lacking *pmrC* (*eptA*) and *cptA*, wild-type, and complemented strains were grown in the presence of 50 μM FeCl_2_ to mid-log phase and normalized to an optical density at 595 nm (OD_595_) of 0.5. A “+” indicates that a strain was transformed with pWSK129 or pACYC184. OMVs were isolated and quantified by measuring protein and lipid content. (A) Protein content of OMVs was measured using the DC assay. Protein concentration was calculated using BSA as a standard. (B) Lipid content of OMV samples was determined by using the lipophilic dye FM 1-43. Values shown are the means ± standard error from three independent experiments. Significance was assessed relative to the wild type using a one-way analysis of variance (ANOVA); ****, *P* ≤ 0.0001.

In C. rodentium and related species, the PmrAB TCS directly regulates transcription of *pmrC* and *cptA*, which are important in maintaining OM integrity and providing resistance to iron and antibiotics (Fig. 1A) (31, 37). OMV production was assessed in C. rodentium Δ*pmrC* (Δ*eptA*) and Δ*cptA* strains grown at various concentrations of FeCl_2_. At each concentration of FeCl_2_, the Δ*pmrC* (Δ*eptA*) Δ*cptA* strain displayed an approximate 2-fold increase in protein and lipid concentrations of OMV preparations, indicating that these genes negatively affect OMV formation (Fig. 3 and Fig. S2). The Δ*pmrC* (Δ*eptA*) and Δ*cptA* strains each exhibited a 1.5-fold increase in OMV production relative to that of the wild type, indicating that these genes both similarly impact vesicle formation. Overexpression of either *pmrC* (*eptA*) or *cptA* in both wild-type and knockout backgrounds reduced vesicle production below the levels of wild-type and empty vector controls (Fig. 3 and Fig. S2). Together, these results show that OMV production is negatively influenced by the PmrAB-regulated proteins PmrC (EptA) and CptA in response to environmental iron in C. rodentium.

### Environmental iron influences OMV production.

**FIG 4:**
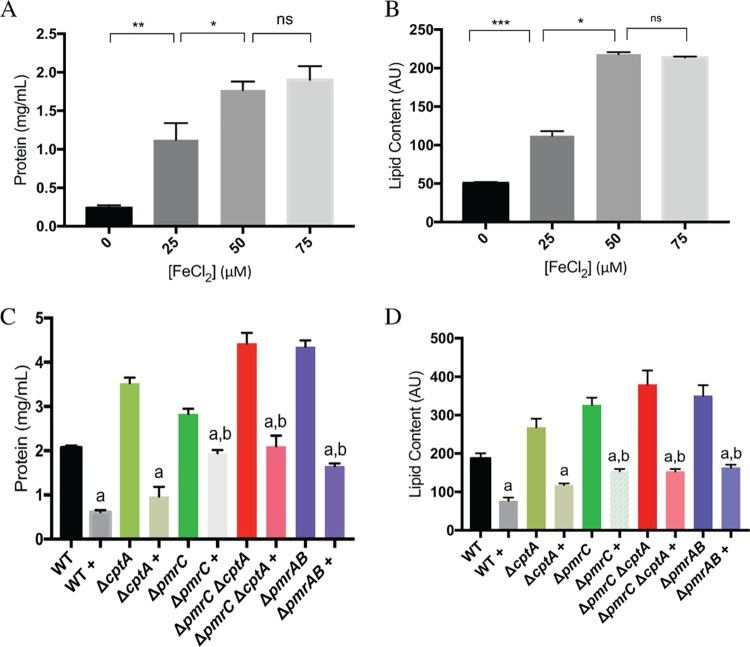
FeCl_2_ increases OMV production in C. rodentium. (A and B) Wild-type C. rodentium strains were grown in various concentrations of FeCl_2_, and OMVs were isolated and quantified. (C and D) OMVs were isolated from various C. rodentium strains grown in 50 μM FeCl_2_ with or without 2 mM glutathione. A “+” indicates that a strain was grown with glutathione. Significance was compared to each strain grown without glutathione (a) and between the wild type grown with glutathione and the mutant strains grown with glutathione (b). Significance between wild-type and mutant strains grown without glutathione is not shown in these graphs. (A and C) Total protein content of OMVs was measured using the DC assay. Protein concentration was calculated using BSA as a standard. (B and D) Lipid content of OMV samples was determined by using the lipophilic dye FM 1-43. Values shown are the means ± standard error from three independent experiments. *, *P* ≤ 0.05; **, *P* ≤ 0.01; ***, *P* ≤ 0.001; ns, not significant.

Since iron activates the PmrAB TCS and PmrAB-controlled genes, including *pmrC* (*eptA*) and *cptA* (31), we investigated the effects of various FeCl_2_ concentrations on OMV production in C. rodentium. OMV formation was assessed in various C. rodentium strains grown at different concentrations of FeCl_2_. Concentrations up to 75 μM FeCl_2_ were used, since the presence of iron at concentrations of 100 μM or higher inhibited bacterial growth due to iron toxicity (31). Unexpectedly, protein and lipid concentrations of OMV samples increased in each C. rodentium strain when grown in the presence of 25 and 50 μM FeCl_2_ (Fig. 4A and B). Increasing the concentration of FeCl_2_ to 75 μM did not greatly increase OMV production (Fig. 4B). Substitution of FeCl_2_ by FeSO_4_ or FeCl_3_ resulted in similar increases in OMV production in wild-type C. rodentium (Fig. S3). These results indicate that environmental iron increases OMV production in C. rodentium. Iron is known to cause oxidative stress by facilitating the production of damaging hydroxyl radicals (35). Furthermore, increased OMV production has been associated with oxidative stress (18). In order to determine whether iron-induced oxidative stress was facilitating OMV formation, we added glutathione to N-minimal medium containing various concentrations of iron and measured OMV production. Glutathione is an antioxidant that can neutralize reactive oxygen species. We therefore grew C. rodentium in the presence of glutathione to neutralize reactive oxygen species to determine if the influence of iron on OMV production is dependent on reactive oxygen species or not.

We found that at each concentration of iron, the presence of 2 mM glutathione reduced OMV formation in various strains (Fig. 4C and D; Fig. S4). This suggests that the iron-mediated increase in OMV formation was due to an increase in reactive oxygen species. In addition, the differences in OMV production between wild-type and Δ*pmrAB* and Δ*pmrC* (Δ*eptA*) Δ*cptA* strains still remained in the presence of glutathione, suggesting that the iron-mediated and the PmrC (EptA) and CptA-mediated responses to OMV formation are likely distinct from one another.

### OMV quantification and size distribution by transmission electron microscopy.

**FIG 5:**
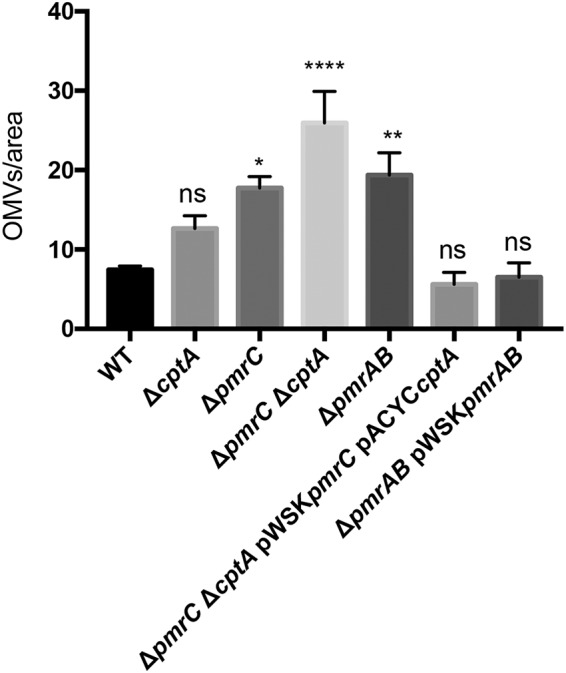
Quantification of OMVs in TEM micrograph images. The number of OMVs counted in each field was divided by the area of the image (square micrometers). Significance shown is relative to the wild type. OMVs were isolated from C. rodentium strains grown to mid-log phase with 50 μM FeCl_2._ Mean quantities of OMVs isolated from each strain were determined by blinded analysis of TEM images using the Macnification software (*n* = 1). Values shown are the means ± standard error. *, *P* ≤ 0.05; **, *P* ≤ 0.01; ****, *P* ≤ 0.0001 (one-way ANOVA).

**FIG 6:**
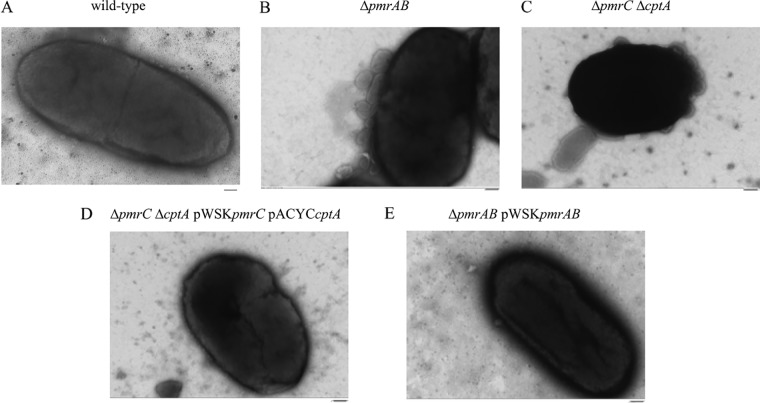
Transmission electron micrograph of C. rodentium cells producing OMVs. C. rodentium wild-type (A), Δ*pmrAB* (B), Δ*pmrC* (Δ*eptA*) Δ*cptA* (C) Δ*pmrC* (Δ*eptA*) Δ*cptA* pWSK*pmrC/eptA* pACYC*cptA* (D), and Δ*pmrAB* pWSK*pmrAB* (E) cells were grown to mid-log phase in N-minimal medium supplemented with 50 μM FeCl_2_. Culture dilutions in PBS were laid onto carbon-coated copper grids and stained as described in Materials and Methods. Samples were imaged using an accelerating voltage of 75 kV and at a magnification of ×40,000. Images shown are representative of 12 micrographs per strain. Bars, 100 nm.

To determine whether the increased OMV production by C. rodentium in the absence of *pmrAB*, *pmrC* (*eptA*), and *cptA* strains is due to either increased number of OMVs or increased vesicle size, OMV samples isolated from cells grown in the presence of 50 μM FeCl_2_ were analyzed by transmission electron microscopy (TEM) (Fig. S5). Consistent with the above data, TEM micrographs revealed an increase in the total number of OMVs produced by strains lacking *pmrAB*, *pmrC* (*eptA*), or *cptA* in comparison to that produced by the wild type (Fig. 5). Complementation-mediated overexpression of *pmrAB* and of both *pmrC* (*eptA*) and *cptA* resulted in a decrease in terms of the numbers of vesicles produced in comparison to that produced by their respective genetic backgrounds (Fig. 5). Furthermore, images of whole cells showed a greater number of vesicles budding from the membranes of the Δ*pmrAB* and Δ*pmrC* (Δ*eptA*) Δ*cptA* strains in comparison to those of the wild-type and complemented strains (Fig. 6). The size distribution of OMVs isolated from each C. rodentium was consistent, as most OMVs were found to be between 20 to 100 nm (Fig. S6). Collectively, these data confirm that the number of OMVs produced by C. rodentium is negatively regulated by the PmrAB TCS and the PmrC (EptA) and CptA proteins.

### C. rodentium OMVs contain the periplasmic β-lactamase.

**FIG 7:**
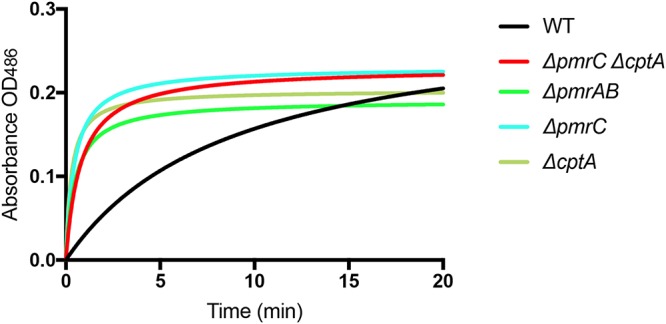
A periplasmic β-lactamase is present in C. rodentium OMVs. OMVs were isolated from various C. rodentium strains grown to mid-log phase with 50 μM FeCl_2._ OMVs were solubilized with 0.1% Triton X-100 and were incubated with the chromogenic substrate nitrocefin. Cleavage of nitrocefin was monitored for 20 min at 22°C by measuring the OD_482_. Data shown are representative of three independent experiments. Data were fitted to a Michaelis-Menten nonlinear regression curve using GraphPad Prism 7 software.

To assess the biological activity of C. rodentium OMVs, the presence of the chromosomally encoded periplasmic β-lactamase (ROD_12321) in OMV preparations was investigated. β-Lactamase activity from OMVs isolated from the C. rodentium wild type, Δ*pmrAB*, Δ*pmrC* (Δ*eptA*) Δ*cptA*, Δ*pmrC* (Δ*eptA*), and Δ*cptA* strains grown in the presence of 50 μM FeCl_2_ was measured using the chromogenic substrate nitrocefin (31). Incubation of equal volumes of solubilized OMVs with nitrocefin resulted in substrate cleavage, indicating the presence of β-lactamase associated with C. rodentium OMVs. In comparison to the wild type, solubilized OMV preparations from the Δ*pmrAB* strain and strains lacking either *pmrC* (*eptA*) or *cptA* resulted in increased β-lactamase activity over 20 min of incubation with nitrocefin (Fig. 7). Given that our sample preparation involved resuspension of OMV with equal volumes, these differences support our earlier findings that OMV production is increased in the absence of *pmrAB*, *pmrC* (*eptA*), and *cptA*. To ensure that differences in activity were not due to changes in β-lactamase expression, periplasm was isolated from each strain, and β-lactamase activity was measured. Using equal volumes of periplasm, no differences in β-lactamase activity were observed between strains, suggesting that there are no differences in cellular expression of β-lactamase between C. rodentium strains (Fig. S7A). Furthermore, to determine whether there was selective enrichment of β-lactamase in OMVs between strains, vesicles isolated from each strain were normalized to the same concentration of total protein. There was no difference in β-lactamase between normalized OMVs isolated from each strain, indicating that the same relative amounts of β-lactamase are loaded into OMVs in each C. rodentium strain (Fig. S7B).

### C. rodentium OMVs contain the outer membrane protease CroP.

**FIG 8:**
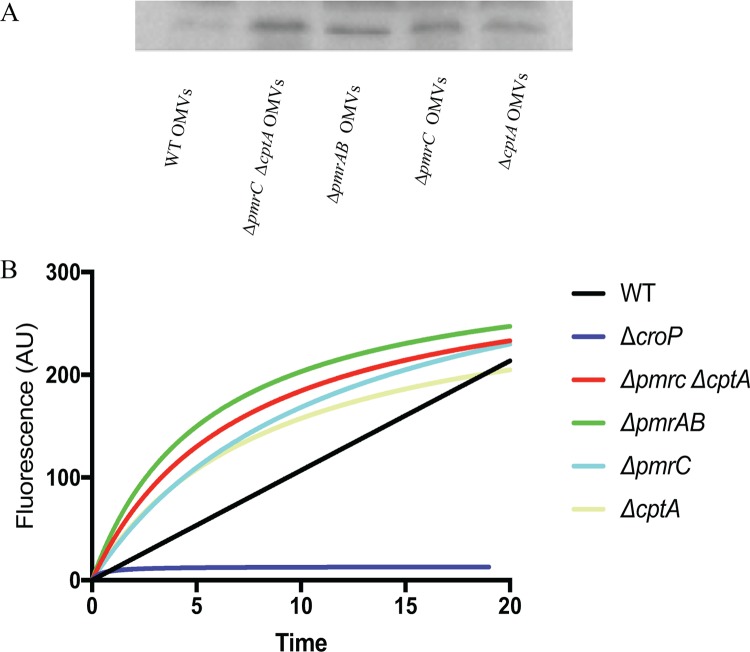
The outer membrane protease CroP is present in C. rodentium OMVs. OMVs were isolated from C. rodentium strains grown to mid-log phase in the presence of 50 μM FeCl_2_. (A) The relative amounts of CroP found within equal volumes of isolated OMVs were determined by Western blotting using an anti-CroP antibody. (B) CroP activity of OMVs were assessed by incubating equal volumes of OMVs isolated from different C. rodentium strains with the C2 FRET substrate for 20 min. Cleavage of the FRET substrate was determined by measuring relative fluorescence at 430 nm after excitation at 325 nm. Data shown are representative of three independent experiments. Data were fitted to a Michaelis-Menten nonlinear regression curve using GraphPad Prism 7 software.

To further assess the biological activity of C. rodentium OMVs, the presence of CroP associated with OMVs was measured. The relative amounts of CroP associated with equal volumes of OMVs from C. rodentium strains grown in the presence of 50 μM FeCl_2_ was determined by Western blotting. As estimated by measuring the intensity of the bands, the amount of CroP was approximately 2- to 3-fold higher in equal volumes of OMVs isolated from strains lacking *pmrAB*, *pmrC* (*eptA*), and *cptA* in comparison to that of the wild type, reflecting the higher number of OMVs produced by these mutated strains (Fig. 8A). Activity of CroP in OMV preparations was measured by incubating equal volumes of OMVs isolated from the C. rodentium strains with the second complement component (C2) fluorescence resonance energy transfer (FRET) substrate (38). In comparison to that of the wild type, equal volumes of OMVs isolated from the Δ*pmrAB*, Δ*pmrC* (Δ*eptA*) Δ*cptA*, Δ*pmrC* (Δ*eptA*), and Δ*cptA* strains resulted in a faster increase in fluorescence over 20 min of incubation, indicating increased CroP activity (Fig. 8B). OMVs isolated from the C. rodentium Δ*croP* strain produced minimal fluorescence, indicating that most of the substrate cleavage is mediated by the CroP protease (Fig. 8B). Furthermore, we confirmed that these differences were due to increased OMV production and not changes in CroP expression or selective enrichment of OMVs, since CroP activity in whole cells and in OMVs normalized to the same protein concentration yielded similar activity curves across different C. rodentium strains (Fig. S8). Together, these results show that OMV preparations from C. rodentium contain biologically active CroP. Consistent with the TEM results, these data confirm that *pmrAB*, *pmrC* (*eptA*), and *cptA* negatively influence OMV production in C. rodentium.

## DISCUSSION

The OM of Gram-negative bacteria is constantly remodeled in response to changing environments sensed by TCS. Covalent modifications of LPS are viewed as an adaptive response to acidic pH, presence of AMPs, and changing concentrations of divalent cations (23). Several enteric bacteria modify the lipid A and core regions of LPS with pEtN modifications. These modifications are primarily catalyzed by the pEtN transferases PmrC (EptA) and CptA, the expression of which is regulated by the PmrAB TCS. Previously, we have shown that PmrC (EptA) and CptA confer protection against iron toxicity and contribute to the maintenance of OM integrity in C. rodentium (31). In the present study, we provide evidence that these proteins negatively affect OMV production in C. rodentium. In the absence of *pmrAB*, *pmrC* (*eptA*), or *cptA*, the production of OMVs is markedly enhanced during the adaptive response to environmental iron (Fig. 2, 3, 5 and 8). These data support the emerging hypothesis that LPS remodeling impacts OMV biogenesis by either enhancing or reducing OMV production in response to changing environmental cues (21, 32).

Iron-induced oxidative stress is one form of envelope stress. Iron induces the formation of reactive oxygen species that damage the envelope of Gram-negative bacteria (35). In the C. rodentium wild type, we found that OMV production is increased when cells are grown in the presence of up to 50 μM FeCl_2_ (Fig. 4). Our data also suggest that this iron-mediated induction of OMV formation is due to increased production of reactive oxygen species. By adding exogenous glutathione, which is an antioxidant, we found that there was reduced OMV formation at 50 μM FeCl_2_ (Fig. 4). Along the murine digestive tract, the concentration of free soluble iron has been estimated to be approximately 50 μM (39). Thus, during colonization of the murine gastrointestinal tract, C. rodentium is likely to encounter concentrations of iron that may induce the formation of OMVs *in vivo*. Increasing the concentration of FeCl_2_ to 75 μM did not yield a subsequent increase in OMV production for wild-type C. rodentium (Fig. 4). Further increasing the concentration of FeCl_2_ was toxic to C. rodentium (data not shown). To our knowledge, the present study is the first that shows the iron-mediated increase in OMV production. These findings are counterintuitive, considering that *pmrC* (*eptA*) and *cptA*, which negatively affect OMV production, are transcriptionally activated in the presence of iron. However, our data suggest that the PmrC (EptA) and CptA- mediated influence on OMV production is independent from the iron-mediated effects of oxidative stress, since differences in OMV production between wild-type and Δ*pmrC* (Δ*eptA*) Δ*cptA* strains were still observed when grown in the presence of the antioxidant glutathione (Fig. 4C and D).

In this study, we demonstrate that OMV production is enhanced in C. rodentium in the absence of *pmrAB*, *pmrC* (*eptA*), and *cptA* (Fig. 2, 3, 5 and 8). Complementation-mediated overexpression with plasmids containing *pmrAB*, *pmrC* (*eptA*), and *cptA* drastically reduced OMV production below wild-type levels (Fig. 2 and 3). We had previously showed that complementation of the Δ*pmrC* (Δ*eptA*) Δ*cptA* strain with either pWSK*pmrC/eptA* or pWSK*cptA* increases resistance to iron toxicity and antibiotics (31). Collectively, these data indicate that *pmrC* (*eptA*) and *cptA* are responsible for maintaining OM integrity and negatively affecting OMV production, suggesting that both processes are required for C. rodentium resistance to iron toxicity. Given that the PmrAB TCS directly regulates *pmrC* (*eptA*) *and cptA* expression (31), our data also support the notion that the increased OMV production observed in the C. rodentium Δ*pmrAB* strain is due to the downregulation of the *pmrC* (*eptA*) and *cptA* genes. Although it remains possible that other PmrAB-regulated genes contribute to the increase in OMV production, most of the observed effects are likely mediated by *pmrC* (*eptA*) and *cptA*, since no significant difference in OMV production was observed when comparing the Δ*pmrC* (Δ*eptA*) Δ*cptA* and Δ*pmrAB* strains.

Our group has previously established the role of PmrC (EptA) and CptA in C. rodentium (31). In other related enteric bacteria, homologs of these proteins function as pEtN transferases. In C. rodentium, a BLAST search revealed that PmrC (EptA) is 92% similar to its homolog in E. coli K-12 MG1655 and 91% similar to its homolog in Salmonella enterica serovar Typhimurium LT2 based on amino acid similarity scores. CptA in C. rodentium is 94% similar to its homolog in E. coli K-12 MG1655 and is 93% similar to its homolog in S. Typhimurium LT2 (40–42). The large amount of amino acid similarity shared between these bacteria suggests that PmrC (EptA) and CptA function similarly as pEtN transferases. However, structural analysis has yet to be performed to confirm that PmrC (EptA) and CptA definitively modify LPS with pEtN moieties.

To assess putative biological functions of C. rodentium OMVs, we tested for the presence of the periplasmic enzyme β-lactamase and the OM protease CroP, associated with OMV preparations. Activity assays showed that both enzymes were associated with OMV preparations isolated from the wild-type, Δ*pmrAB* and Δ*pmrC* (Δ*eptA*) Δ*cptA* strains (Fig. 7 and 8). Consistent with the higher numbers of OMVs produced by the deletion mutants (Fig. 4 and 5), OMVs resuspended with the same volumes isolated from the Δ*pmrAB* and Δ*pmrC* (Δ*eptA*) Δ*cptA* strains exhibited higher β-lactamase and CroP enzymatic activities than an equal volume of OMVs isolated from the wild-type strain.

In a recent study, Elhenawy et al. showed that the PhoPQ-regulated PagL lipid A deacylase enhances OMV formation in S. enterica (32). The C. rodentium genome does not contain the *pagL* gene; however, it is possible that other PhoPQ-regulated LPS-modifying enzymes fulfill a similar role in enhancing OMV production. In the current study, we show that PmrC (EptA) and CptA repress OMV formation in C. rodentium. Together, these studies might indicate that different LPS modifications have the ability to either enhance or repress OMV production.

Confirming the functional role of CptA and PmrC (EptA) in C. rodentium will help us understand how these proteins affect OMV production and/or membrane integrity in the presence of iron induced-stress. If these proteins are indeed pEtN transferases, as is the case in E. coli and Salmonella enterica, the decrease in OMV production may be a response to maintain pEtN-modified LPS in the OM, and, in turn, strengthen OM integrity (21, 29, 30, 40, 43). Furthermore, the role of CptA and PmrC (EptA) as pEtN transferases that repress OMV production is consistent with the previously proposed model for regulation of membrane lipid (21, 30, 32). As pEtN is added to LPS, this may minimize membrane curvature and subsequent OMV formation. Together, our data are the first to show that PmrC (EptA) and CptA effect OMV production induced by iron-dependent environmental stress.

## MATERIALS AND METHODS

### Bacterial strains, growth conditions, and reagents.

All bacterial strains and plasmids used in this study are listed in Table 1. Bacteria were grown overnight in Luria-Bertani (LB) broth at 37°C with aeration. Bacteria were diluted 1:100 in N-minimal medium [50 mM Bis-Tris (pH 7.5), 7.5 mM (NH_4_)SO_4_, 5 mM KCl, 0.5 mM K_2_SO_4_, 0.5 mM KH_2_PO_4_, 38 mM glycerol, and 0.1% (wt/vol) Casamino Acids] supplemented with 0.2% glucose, 20 μM MgCl_2_, and various concentrations of FeCl_2_ and glutathione, as indicated. Cultures were grown statically for 48 h at 28°C, leading to an optical density at 595 nm (OD_595_) of approximately 0.5. When appropriate for plasmid selection, kanamycin (Kan; 50 μg/ml) and chloramphenicol (Cm; 35 μg/ml) were added to the medium.

**TABLE 1:** Bacterial strains and plasmids used in this study

Strain or plasmid	Description^*a*^	Reference or source
C. rodentium strains		
DBS100	Wild type	46
Δ*pmrAB*	DBS100 Δ*pmrAB*	31
Δ*pmrc* Δ*cptA*	DBS100 Δ*pmrC* Δ*cptA*	31
Δ*pmrC*	DBS100 Δ*pmrC*	31
Δ*cptA*	DBS100 Δ*cptA*	31
Δ*croP*	DBS100 Δ*croP*	47
Plasmids		
pWSK129	Low-copy-number cloning vector, Kan^r^	48
pACYC184	Low-copy-number cloning vector, Cm^r^	NEB
pWSK*pmrC*	*pmrC* cloned into pWSK129 (native promoter)	31
pWSK*cptA*	*cptA* cloned into pWSK129 (native promoter)	31
pACYC*cptA*	*cptA* cloned into pACYC184 (native promoter)	This study
pWSK*pmrAB*	*pmrAB* cloned into pWSK129 (under control of constitutive promoter at the 3′ end of the *pmrC* coding region)	31

a Kan^r^, kanamycin resistance; Cm^r^, chloramphenicol resistance; NEB, New England BioLabs.

### OMV isolation.

OMVs were isolated as previously described (21). Briefly, 50 ml of each bacterial culture was normalized to an OD_595_ of 0.5 and centrifuged (12,000 × *g*, 4°C, 20 min). Cultures were grown for 48 h in order to obtain a robust and quantifiable amount of OMVs. Supernatants were harvested and sequentially filtered through 0.8-μm and 0.45-μm membrane filters (Millex, Millipore). The resulting filtrates were concentrated using a 10-kDa molecular weight cutoff centrifugal filter unit (Amicon Ultra-15, 2,360 × *g*, 4°C, 20 min; Millipore). Concentrated filtrates were then centrifuged (200,000 × *g*, 4°C, 2.5 h) in a tabletop Optima ultracentrifuge (Beckman Coulter). Pelleted OMVs were resuspended in 300 μl of phosphate-buffered saline (PBS).

### OMV protein quantification.

Protein concentrations of OMV preparations were determined by using the detergent-compatible (DC) protein assay (Bio-Rad) (44). OMVs were solubilized with 0.1% Triton X-100 (4°C, 15 min), and the DC protein assay was performed according to the manufacturer’s instructions. The OD_750_ was measured using a Hitachi U-2010 spectrophotometer. Protein concentrations were determined using bovine serum albumin (BSA) as a standard. In experiments in which OMVs were normalized to the same protein concentrations, all purified OMV samples were normalized to a protein concentration of 1.5 mg/ml.

### OMV lipid quantification.

OMV samples were quantified using the lipophilic dye FM 1-43 [*N*-(3-triethylammoniumpropyl)-4-(4-(dibutylamino) styryl) pyridinium dibromide; Molecular Probes, Life Technologies], which provides an indirect measurement of lipid content, as shown previously (45). OMV samples were diluted 1:55 in PBS and transferred to a quartz cuvette. The FM 1-43 dye was added at a final concentration of 4.5 μM. Following excitation at 479 nm, fluorescence emission at 600 nm was measured on a Varian Cary Eclipse fluorescence spectrophotometer, using 5-nm slit widths for both excitation and emission.

### TEM.

For bacterial cell imaging, culture aliquots were transferred onto carbon-coated copper grids and grown for an additional 24 h. Grids were fixed with 2.5% glutaraldehyde diluted in 50 mM sodium cacodylate (pH 7.2) and washed 3 times with 3% saccharose diluted in the same buffer. For OMV imaging, purified OMV samples were diluted 1:20 with Dulbecco’s modified Eagle medium (DMEM), and 70 μl aliquots were laid onto carbon-coated copper grids by ultracentrifugation (Airfuge; 20,000 lb/in^2^, 5 min). Excess liquid was discarded from the grids and samples were negatively stained with phosphotungstic acid (3% [wt/vol]) for 5 min and dried with filter paper. Specimens were examined blinded under a microscope (H-7100; Hitachi) operated at an accelerating voltage of 75 kV and at magnifications of ×40,000 for cells and ×30,000 for OMVs. The size and number of OMVs per micrograph were determined blinded using the Macnification software (Orbicular).

### Nitrocefin assays.

The activity of β-lactamase in OMVs and periplasm was determined by measuring cleavage of the chromogenic β-lactam nitrocefin (31). OMVs were solubilized with 0.1% Triton X-100 for 15 min at 4°C. Enzymatic assays were performed at 22°C in the presence of 43 μM nitrocefin, and β-lactamase activity was monitored by measuring the OD_486_ for 45 min, using a Powerwave X340 microplate reader (Bio-Tek instruments). Data were fitted to a Michaelis-Menten nonlinear regression curve using GraphPad Prism 7 software.

### Periplasmic protein extraction.

C. rodentium cells were grown to an OD_595_ of 0.5 in N-minimal medium and centrifuged (12,000 × *g*, 4°C, 20 min). Cell pellets were resuspended with periplasting lysis buffer (1 M Tris HCl, 20% sucrose, 1 mM EDTA, 40 U/μl lysozyme) and incubated at 22°C for 5 min. Purified water was added to the lysed cells, which were then incubated for 10 min at 4°C. Lysed cells were then centrifuged (4,000 × *g*, 22°C, 20 min) to extract the supernatant containing periplasmic proteins.

### Western blotting.

Purified OMVs (10 μl) were added to Laemmli sample buffer, boiled for 5 min, and resolved on a 10% SDS-PAGE gel. Proteins were transferred to a polyvinylidene difluoride membrane and Western blotting was performed using a rabbit polyclonal antibody raised against CroP and a peroxidase-conjugated anti-rabbit secondary antibody, as previously described (38). Quantity One software (Bio-Rad) was used to image and quantify protein bands.

### CroP activity assays.

The activity of the outer membrane protease CroP in OMVs or whole C. rodentium cells was determined by measuring the cleavage of the C2 FRET substrate [2Abz-SLGRKIQIK(Dnp)-NH_2_; AnaSpec], as previously described (38). OMVs and C. rodentium cells grown to an OD_595_ of 0.5 were diluted in PBS, transferred into a quartz cuvette, and incubated with the FRET substrate (6 μM) at 22°C. Cleavage of the FRET substrate was monitored over 60 min by measuring fluorescence emission at 430 nm at an excitation wavelength of 325 nm using a Varian Cary Eclipse fluorescence spectrophotometer. Excitation and emission slit widths were set at 5 nm. Data were fitted to a Michaelis-Menten nonlinear regression curve using GraphPad Prism 7 software.

## Supplementary Material

Supplemental file 1
