# Supplementary material for: PmrC (EptA) and CptA Negatively Affect Outer Membrane Vesicle Production in Citrobacter rodentium
Source: J Bacteriol. 2019 Mar 13;201(7):e00454-18. doi: 10.1128/JB.00454-18 (PMC6416907; doi:10.1128/JB.00454-18)
Supplement: Supplemental file 1 [file JB.00454-18-s0001.pdf]

Supplementary Figures:

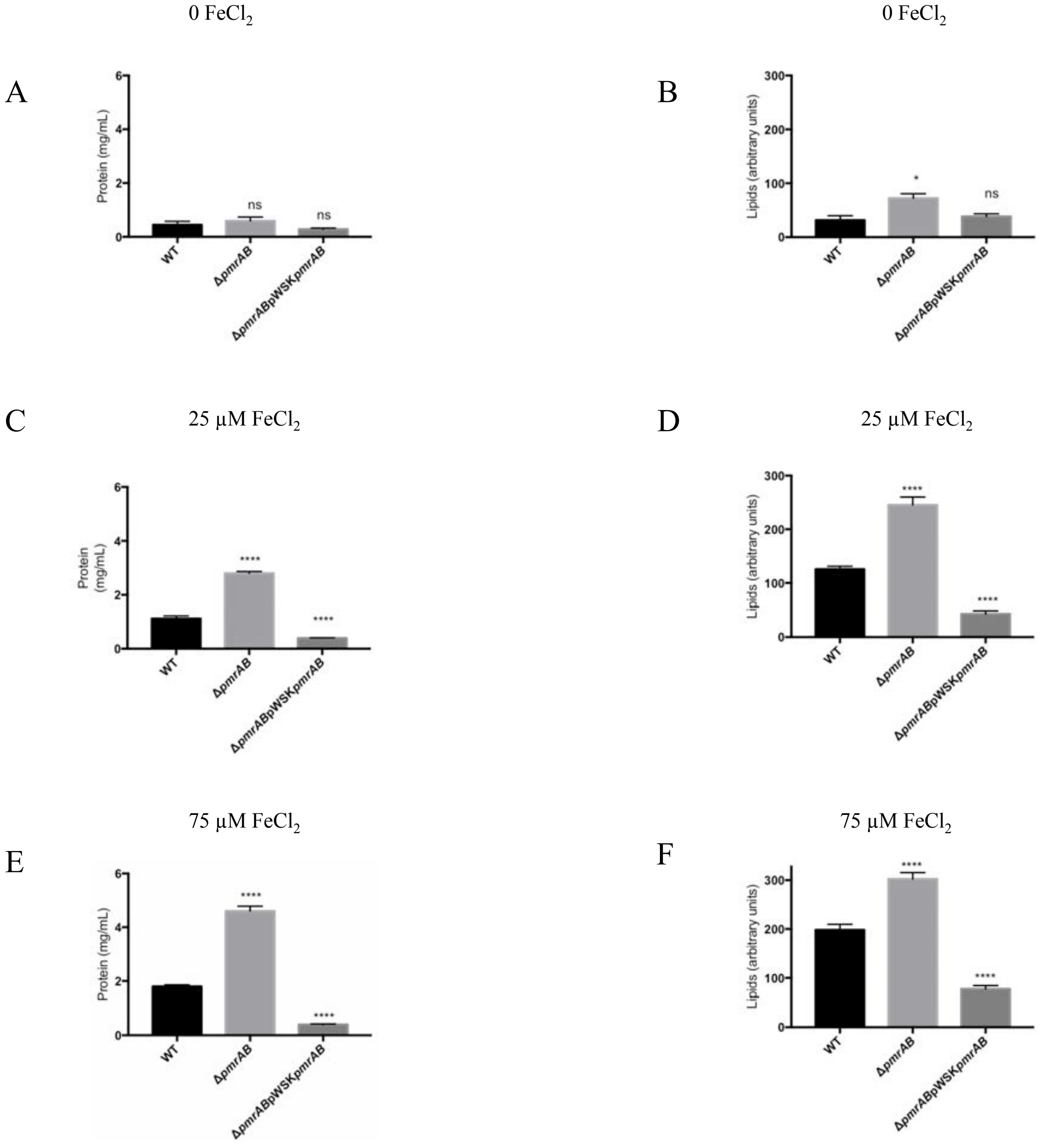

Fig S1 Deletion of the *pmrAB* TCS genes results in increased OMV biogenesis in *C. rodentium* at various concentrations of $FeCl_2$. OMVs isolated from the *C. rodentium* wild-type, Δ*pmrAB*, and Δ*pmrAB* pWSK*pmrAB* strains grown with either (A-B) no $FeCl_2$, (C-D) 25 µM $FeCl_2$, or (E-F) 75 µM $FeCl_2$ were quantified by measuring protein and lipid contents. (A,C,E). Protein content of OMVs was measured using the DC protein assay. Protein concentration was calculated using BSA as a standard. (B,D,F) Lipid content of OMV samples was determined by using the lipophilic dye, FM 1-43. Cultures were grown to mid-log phase and normalized to an OD_{595} of 0.5 prior to OMV isolation. Values shown are the means ± standard error from three independent experiments. Significance was assessed relative to wild-type using one-way ANOVA, **** $P \leq 0.0001$.

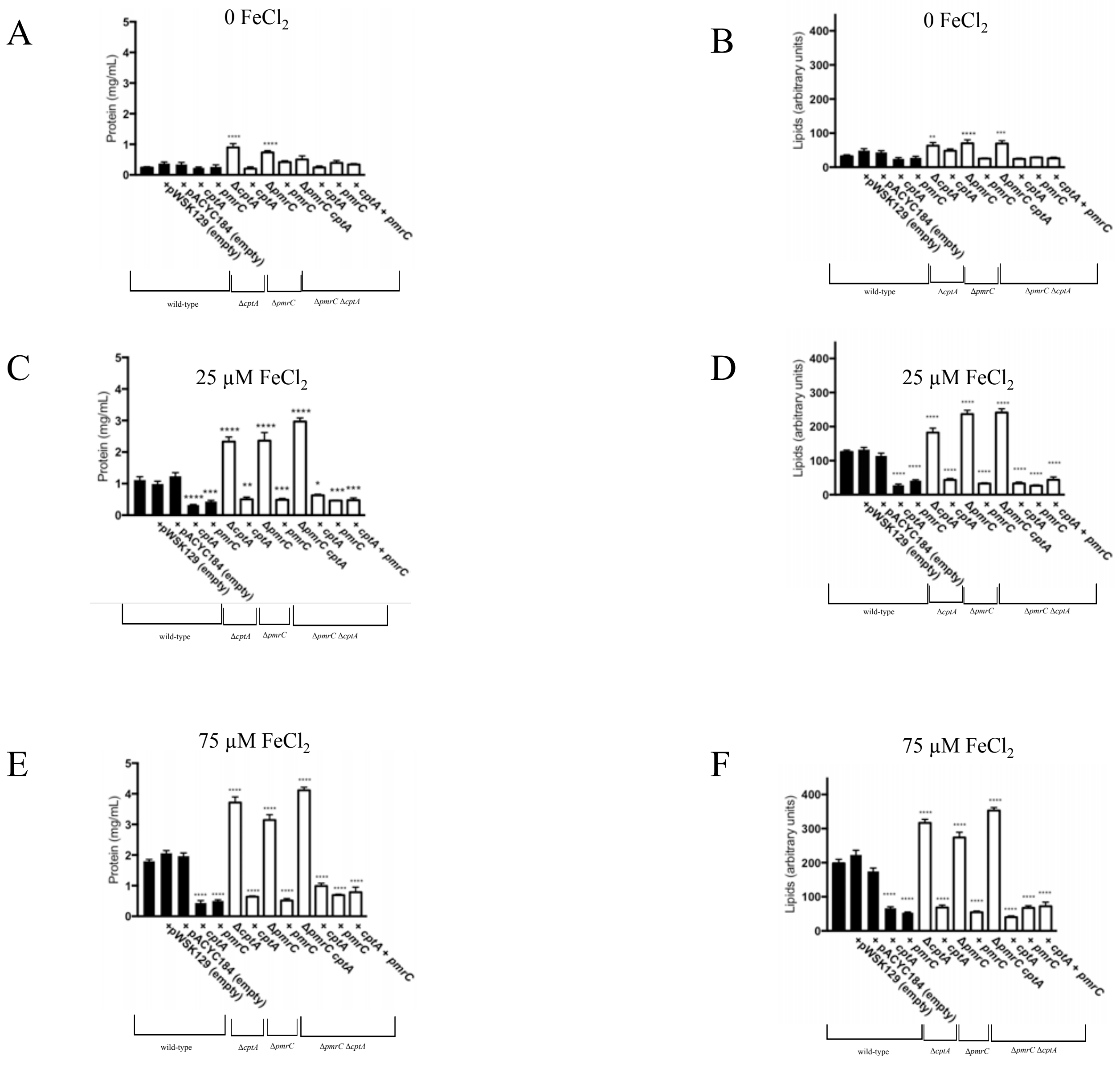

Fig S2 Deletion of *pmrC* and *cptA* results in increased OMV biogenesis in *C. rodentium* at various concentrations of $FeCl_2$. *C. rodentium* strains lacking *pmrC* and *cptA*, wild-type, and complemented strains were grown with either (A-B) no $FeCl_2$, (C-D) 25 µM $FeCl_2$, or (E-F) 75 µM $FeCl_2$ to mid-log phase and normalized to an OD_{595} of 0.5. Kanamycin (50 µg/ml) and chloramphenicol (35 µg/ml) were added during growth when plasmid selection was appropriate. "+" indicates when a strain was complemented with pWSK129 or pACYC184. OMVs were isolated and quantified by measuring total protein and lipid content. (A,C,E) Protein content of OMVs was measured using the DC assay. Protein concentration was calculated using BSA as a standard. (B,D,F) Lipid content of OMV samples was determined by using the lipophilic dye, FM 1-43. Values shown are the means ± standard error from three independent experiments. Significance was assessed relative to wild-type using a one-way ANOVA, , * $P \leq 0.05$, ** $P \leq 0.01$ **** $P \leq 0.0001$.

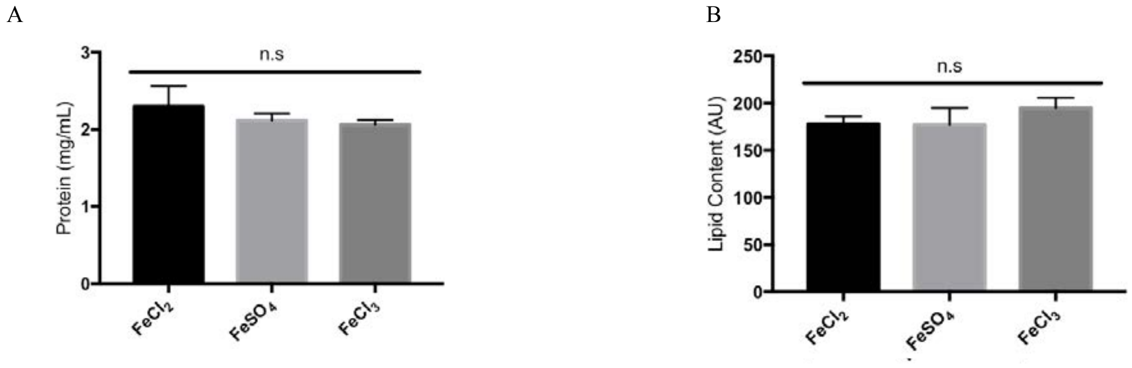

Fig S3 OMV production in wild-type *C. rodentium* grown in different iron sources. OMVs were isolated from wild-type *C. rodentium* grown with 50 µM of either $FeCl_2$, $FeCl_3$, or $FeSO_4$. (A) Protein content of OMVs was measured using the DC assay. Protein concentration was calculated using BSA as a standard (B) Lipid content of OMV samples was determined by using the lipophilic dye, FM 1-43. Significance show is relative to wild-type. Values shown are the means ± standard error from a single independent experiment (n=1), repeated in triplicate, one-way ANOVA.

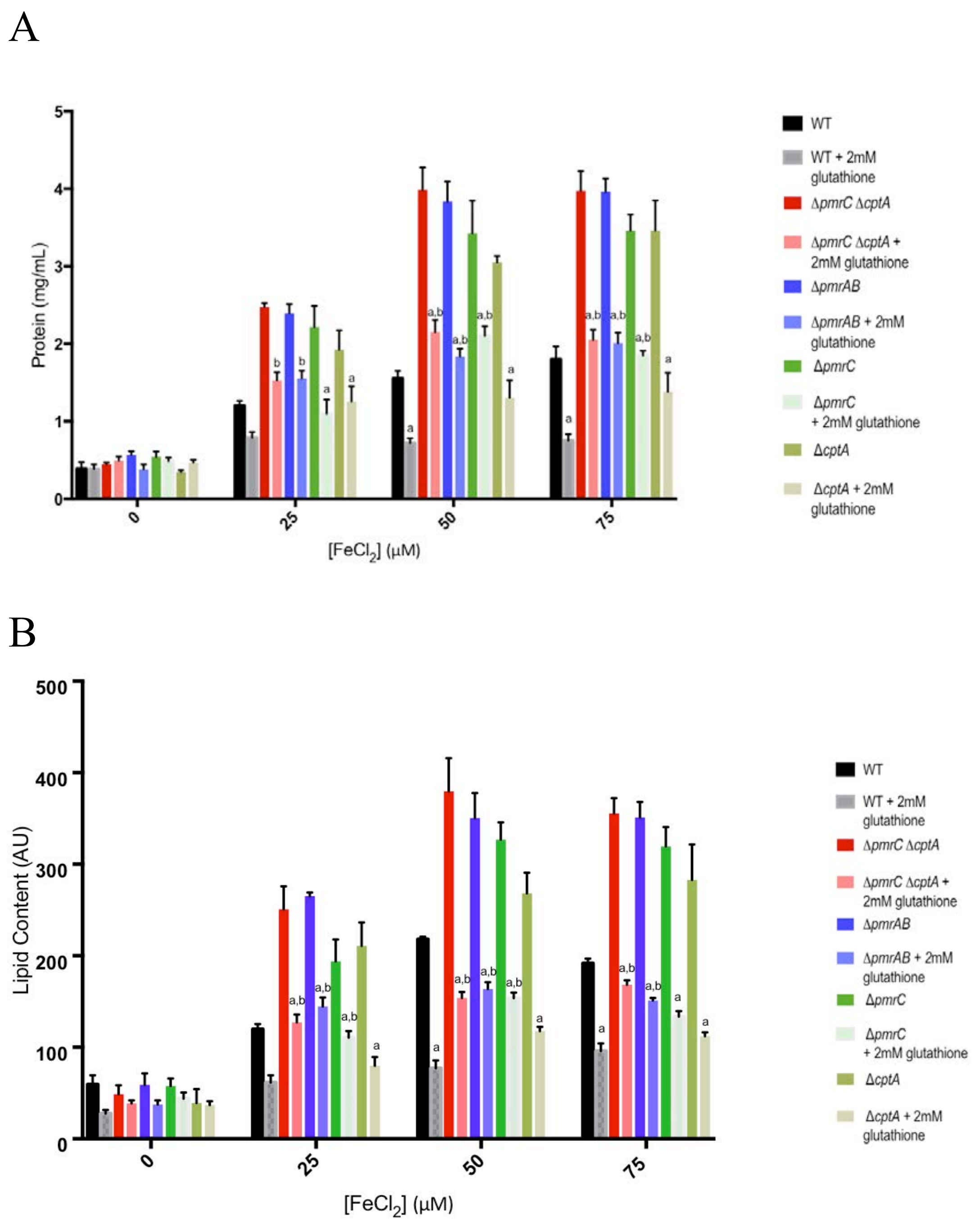

Fig S4 Glutathione decreases OMV production in the presence of iron. OMVs were isolated from various *C. rodentium* strains grown in 50 μM $FeCl_2$ with or without 2mM glutathione. A) OMVs were quantified by measuring protein and lipid content. Protein content of OMVs was measured using the DC assay. Protein concentration was calculated using BSA as a standard. (B) Lipid content of OMV samples was determined by using the lipophilic dye, FM 1-43. At each given concentration of $FeCl_2$, significance was compared to each strain grown without glutathione (shown by "a") and between wild-type grown with glutathione and the mutant strains grown with glutathione (shown by "b"). Significance between wild-type and mutant strains grown without glutathione are not shown in these graphs. Values shown are the means ± standard error from a single independent experiment (n=1), repeated in triplicate, one-way ANOVA. Significance: $P \leq 0.05$.

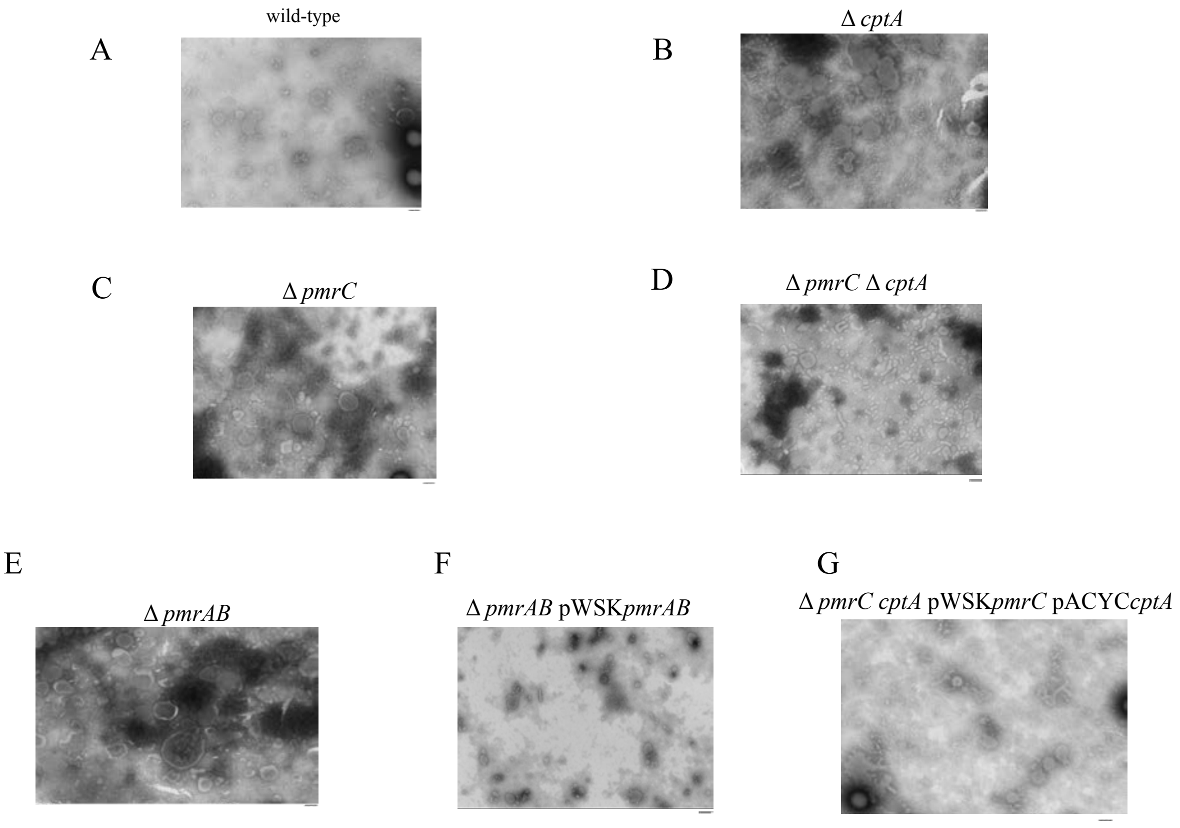

Fig S5 TEM of *C. rodentium* OMVs. OMV samples were laid onto carbon-coated copper grids and stained as described in the Materials and Methods section. Transmission electron microscopy of specimens was conducted using an accelerating voltage of 75 kV and at a magnification of 30,000 x. Images shown are representative of 10 different fields for OMVs isolated from each strain, bars 100 nm.

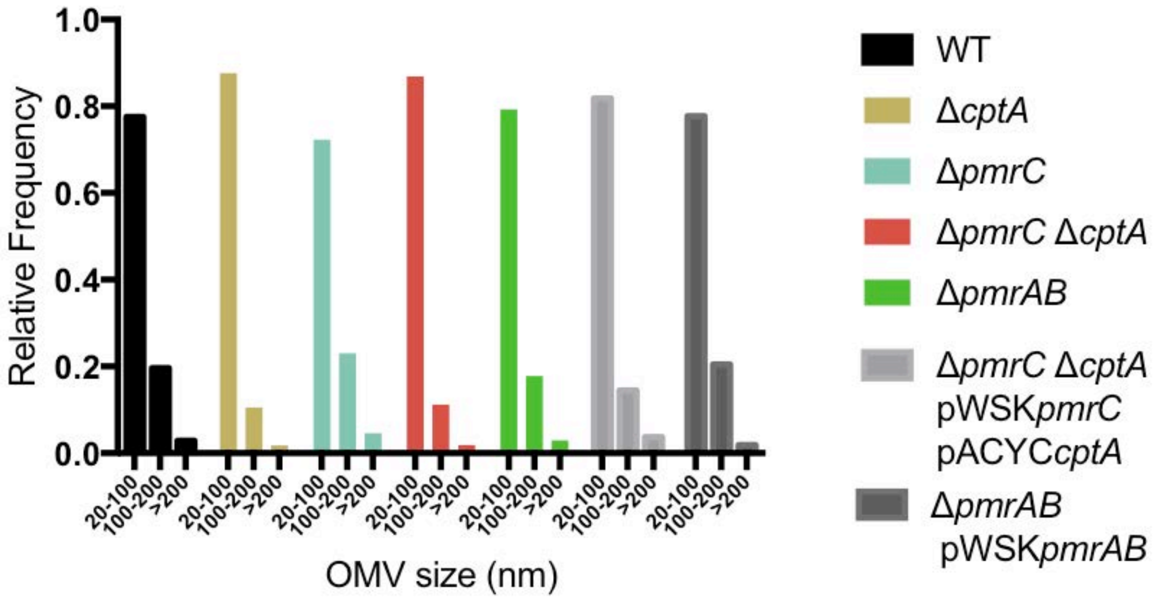

Fig S6 Size distribution of *C. rodentium* OMVs The size distribution of OMVs from different *C. rodentium* strains was determined by relative frequency. OMVs were isolated from *C. rodentium* strains grown to mid-log phase with 50 µM $FeCl_2$.. The mean size of OMVs isolated from each strain were determined by blinded analysis of TEM images using the Macnification software (n=1).

A B

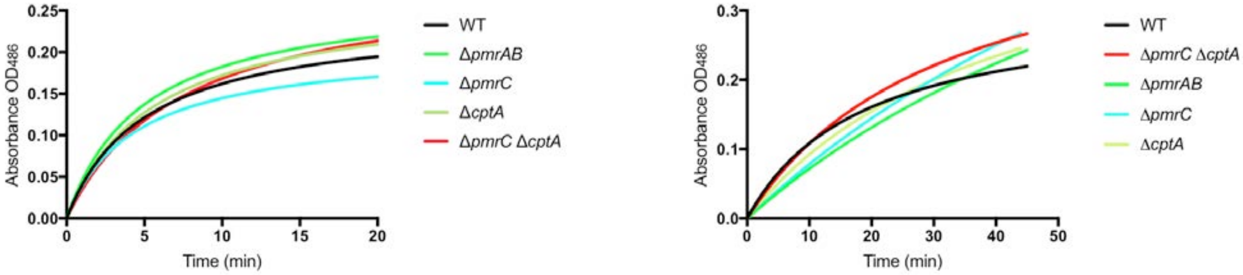

Fig S7 *C. rodentium* strains produce similar amounts of ß-lactamase OMVs and periplasmic extracts were isolated from various *C. rodentium* strains grown to mid-log phase with 50 µM $FeCl_2$.. A) Periplasmic proteins were extracted from whole *C. rodentium* cells using a periplasting buffer. B) OMVs were normalized to a total protein concentration of 1.5 mg/mL and were solubilized with 0.1% Triton-X-100 and incubated with the chromogenic substrate, nitrocefin for 20 min. Equal volumes of extracts were incubated with the chromogenic substrate, nitrocefin for 45 min. Cleavage of nitrocefin was monitored at 22 °C by measuring the OD_{482}. Data shown are representative of three independent experiments. Data were fit to a Michaelis-Menten nonlinear regression curve using the GraphPad Prism 7 software.

A

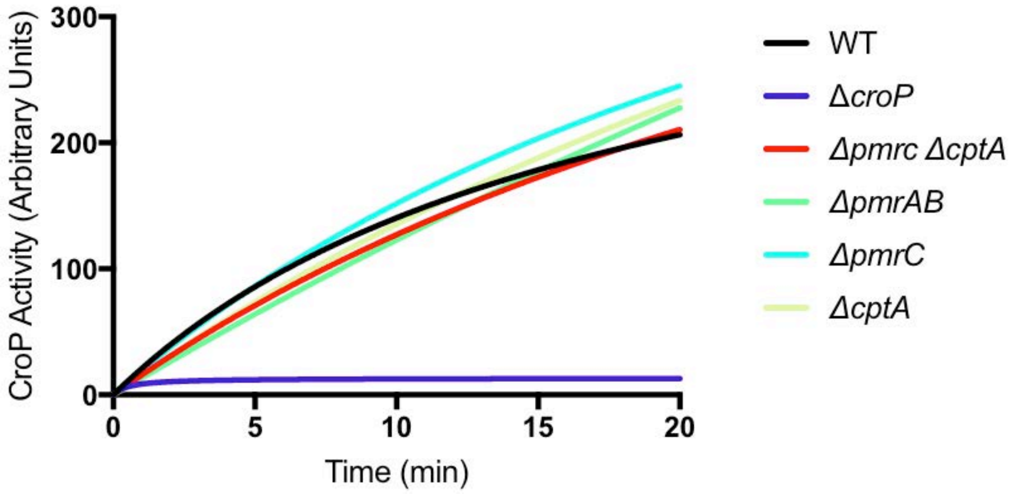

B

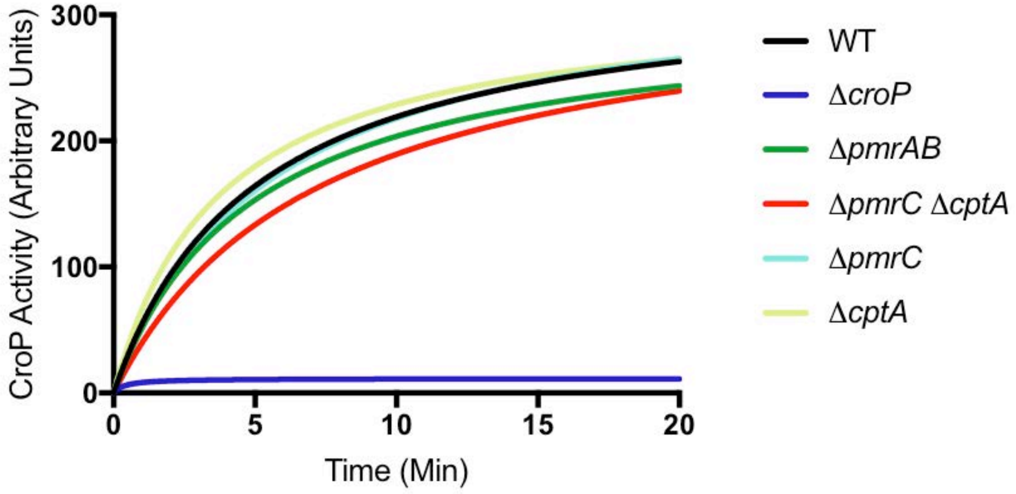

Fig S8 *C. rodentium* strains produce similar amounts of CroP. CroP activity was assessed from (A) whole cells grown to mid-log phase and normalized to an OD_{595} = 0.5 and (B) OMVs isolated from different *C. rodentium* strains normalized to a total protein concentration of 1.5 mg/mL. OMVs and cells were incubated with the C2 FRET substrate for 20 min. Cleavage of the FRET substrate was determined by measuring relative fluorescence at 430 nm after excitation at 325 nm. Data shown are representative of three independent experiments. Data were fit to a Michaelis-Menten nonlinear regression curve using the GraphPad Prism 7 software.
